# Neurological consequences of climate change: a review of emerging challenges and potential impacts on brain health

**DOI:** 10.1097/MS9.0000000000003425

**Published:** 2025-05-26

**Authors:** Syed Daniyal Ahmed Jilanee, Muhammad Saeed, Muhammad Umar Ahsan, Muhammad Usman Farooq, Somaiya Ahmed, Hiba Shahid, Sajawal Sharif

**Affiliations:** a Liaquat National Hospital and Medical College, Karachi, Pakistan; b D.G. Khan Medical College, Dera Ghazi Khan, Pakistan; c Sir Salimullah Medical College, Dhaka, Bangladesh; d Ziauddin University, Karachi, Pakistan

**Keywords:** brain health, climate change, environmental factors, heat-related diseases, neurodegeneration

## Abstract

Climate change causes significant challenges to the neurological system due to both gradual and immediate environmental changes. This paper explores the various issues facing by the brain because of climate change, such as increased cases of heat-related diseases, neuroinflammation, oxidative stress, altered patterns of diseases, and phenomena associated with very severe weather conditions. A comprehensive literature search was conducted. This paper demonstrates evidences, linking climate-related factors (air pollution, heat exposure, and vector-borne diseases) to neuroinflammation and oxidative stress, the major contributors to neurodegenerative diseases. Additionally, it addresses targeted interventions to mitigate health risks, particularly among vulnerable groups such as the elderly and children. Moreover, it was noticed how climate change affects mental health in terms of anxiety and depression. Chronic stress disorders should, therefore, be considered during mental health interventions for climate adaptation. The study highly emphasizes collaborative research networks and open-access data repositories while advocating for interdisciplinary approaches and policy support to deepen our knowledge and lessen neurological effects related to climatic variability. This review highlights the importance of multidisciplinary research and policy interventions to mitigate these risks and protect global neurological health.

## Introduction

### Brief overview of climate change

Long-standing shifts in temperature, the pattern of precipitation, and more frequent extreme weather events characterize climate change. It is driven mainly by deforestation, burning fossil fuels, and toxic smoke and waste produced by industries. These activities have considerably increased greenhouse gas concentrations, for instance, carbon dioxide, methane, and nitrous oxide in Earth’s atmosphere, resulting in a warming effect known as global warming^[1]^.

**Figure 1.**
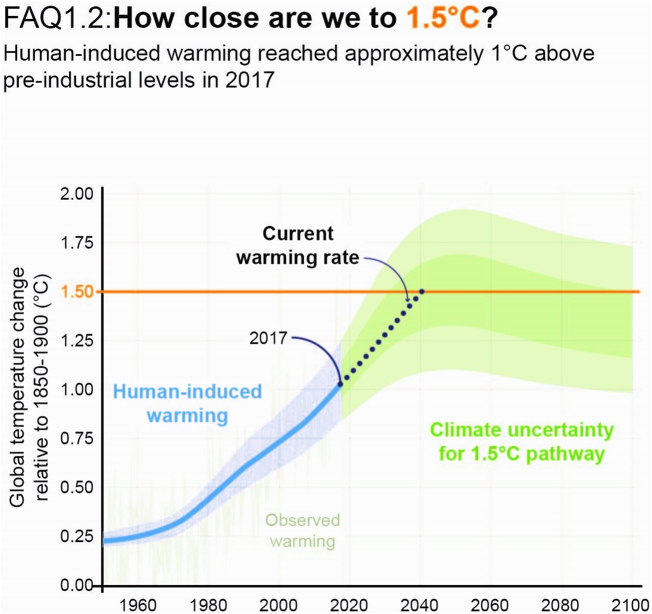
Overview of climate change

According to the Intergovernmental Panel on Climate Change (IPCC), there is a considerable rise in the average global temperature by about 1.5°C, more than what it was before the industrial revolution. Furthermore, the trend is expected to continue leading to severe impacts on both natural ecosystems and humanity^[1]^ (Fig. 1).

**HIGHLIGHTS**

- Climate change poses a significant threat to human health, with its effects extending to the neurological system.
- How climate-related factors, such as air pollution, heat exposure, and vector-borne diseases, contribute to neuroinflammation, oxidative stress, and neurodegenerative diseases.
- The link between climate change and mental health emphasizes the role of chronic stress and anxiety in populations facing the consequences of climate change.
- The importance of collaborative research networks, open-access data repositories, and interdisciplinary approaches to deepen our understanding of the neurological effects of climate change.

### Importance of studying neurological consequences

Climate change has a broad environmental impact, evident in aspects such as rising sea levels, loss of biodiversity, change in agricultural outputs, etc. While climate change’s effects on ecosystems and weather patterns are well-documented, its impact on human health – specifically neurobehavioral changes affecting brain development and disease – remains undiscovered. Extreme weather events, heat waves, and increased pollution have been correlated with some neurological diseases such as stroke, dementia, and mental health problems like anxiety and depression^[2,3]^. Also, climate change and food insecurity can worsen depression and anxiety through climate-induced migration^[2]^.

There are many reasons why knowing these neurological effects is essential. One of them is that it shows the impacts on human health triggered by global warming. Secondly, it helps to highlight groups that need special protection, like the elderly, kids, and people with ailments. A holistic approach emphasizes the association between human and environmental health in addressing climate change^[4]^.

### Purpose of the review

This review aims to traverse existing literature on the neurological impacts of climate change, underscoring emerging challenges and potential effects on brain health and lesser-known ways it impacts brain health like extreme heat and pollutants. It seeks to discover how global warming affects brain health by looking at what has already been discovered about this topic and shedding light on how it can work. This review also highlights gaps in our understanding, proposing future research directions to formulate strategies for and mitigate the neurological effects of a changing climate, and help develop strategies to protect brain health as the environment continues to change.

## Methodology

### Search strategy

We conducted a comprehensive search on various databases including PubMed, Google Scholar, and Scopus from their inception until July 2024. Keywords such as “climate change,” “environmental changes,” “Brain health” and “neurological disorders” were used to identify relevant articles.

### Study selection

Studies were selected on the basis of their title and abstract, and were further discussed before inclusion in this review. Studies were thoroughly reviewed in order to generate a comprehensive narrative review on the topic.

### Inclusion and exclusion criteria

We included studies focusing on: (1) Neurological Consequences of Climate Change, (2) heat-related diseases, (3) Climate related neurological disorders, (4) studies in English language only, (5) clinical trials, observational studies, systematic reviews and meta-analysis, case reports, and case series, and (6) studies published after 2010. Studies were excluded: (1) that were not following the inclusion criteria, (2) studies in languages other than English, and (3) studies published before 2010.

## Neurological health and change of climate

### Effects of environmental factors on neurological health

Environmental factors significantly affect neurological health, including extreme temperatures, air pollution, and contamination by heavy metals and pesticides. Neurological diseases are significantly at risk due to air pollution, including NO_2_, SO_2_, ozone (O_3_), and fine particulate matter (PM2.5). Studies claim that cognitive decline is linked with long-standing air pollution as well as other related syndromes in children, including neurodevelopmental diseases and the occurrence of higher instances of neurodegenerative ailments such as Parkinson’s and Alzheimer’s^[3,5]^.

Early exposure to PM2.5 (particles less than 2.5 micrometers in diameter) can impair a child’s neurodevelopment, resulting in diminished brain hemisphere development patterns, behavioral development, cognitive and psychomotor development, and intellect. A loss of 0.38 points in language function scores at 24 months of a child is linked to every 1 μg/m^3^ increase in prenatal PM2.5 exposure (95% CI: −0.77 to −0.01). The third trimester of pregnancy is when this link is most noticeable^[6]^.

Conditions of extreme temperature and heatwaves caused by a change in climate are also likely to affect our nerves. Increased heat worsens pre-existing neurotic disorders while making one susceptible to attacks like heat stroke or other dehydration incidents caused by excessive temperatures. It has been noticed that besides dehydration, the brain’s circulation might be affected if the environment is extreme^[7]^. Additionally, stressors like heat can also produce inflammatory responses adding to mental health disorders and neurodegeneration^[8]^.

A study by Louis *et al* highlighted that temperature variability accounted for 2%–4% of first-time strokes in China, while heat waves contributed to 72.1% of the cerebrovascular disease disability burden in South Korea. A 1.5°C mean temperature increase was linked to a 12% rise in dementia admissions, with dementia patients at higher risk during extreme heat events.

Higher annual PM2.5 and NO2 levels were associated with increased migraine-specific urgent care visits. Short-term exposure to PM2.5, PM10, NO2, O3, and CO correlated with migraines, and O3 exposure was elevated on days of tension-type headache exacerbations.

**Figure 2.**
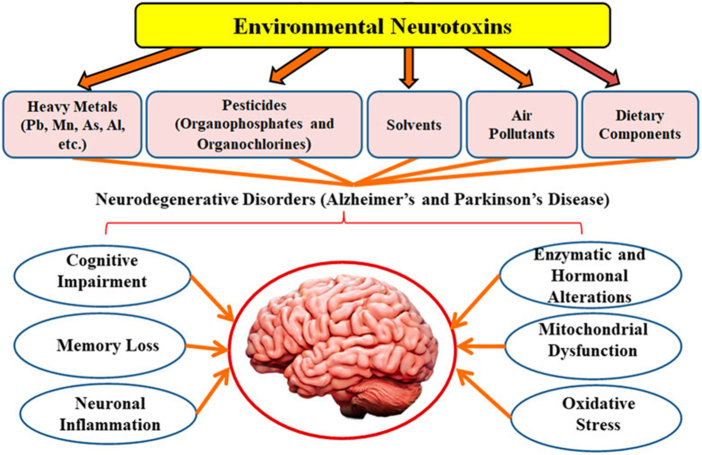
Environmental neurotoxins and neurodegenerative disorders

In Parkinson’s disease (PD), 2-year exposures to PM2.5, NO2, and O3 were associated with increased incidence (HR: 1.03–1.04). However, the link between NO2 and PD remains controversial^[9]^ (Fig. 2).

Other than that, the temperature rise can predispose to a higher frequency of mosquito-related infections especially Zika Virus Infection, West Nile Fever, and Lyme disease which particularly target the Central Nervous System. The diseases result in serious conditions like meningitis, and encephalitis that can lead to abnormalities in motor skills and cognitive disabilities^[7]^.

### Mechanisms linking climate change to neurological consequences

There are several ways in which the change in climate affects neurological health, both directly and indirectly. One of the significant ways is by making pollutants worse. The hotter it gets, the more ozone and PM2.5 there will be at ground level, thus deteriorating the air quality and increasing the amount of substances people breathe in^[10]^. The elements must have the capability to penetrate through the blood-brain barrier, causing neuroinflammation and induced oxidative stress; both of these mechanisms are central during the establishment of neurodegenerative conditions^[11]^.

Particulate matter (PM) exposure raises the synthesis of pro-inflammatory cytokines, including GM-CSF, IL-1β, and IL-6, which are then released into the bloodstream through interactions with airway epithelial cells and alveolar macrophages. This process contributes to oxidative stress and cellular damage by promoting lipid peroxidation, increasing NFκβ expression, and activating MAP kinase signaling, especially through the JNK pathway. Additionally, exposure to PM increases inflammation via circulating neutrophils and monocytes generated from bone marrow. These blood-brain barrier-crossing systemic cytokines, such as TNF-α and IL-1β, cause neurochemical changes, behavioral abnormalities, and neuroinflammation, highlighting the harmful effects of air pollution on brain function^[12]^.

Pollution impacts embryonic neurodevelopment in several ways, such as intrauterine inflammation, which leads to the loss of cells in the central nervous system, PM2.5 exposure during pregnancy may raise oxidative stress in the mother, which raises the production of pro-inflammatory cytokines and causes placental malfunction. Similarly, elevated prenatal oxidative stress generates cytokines that influence several neurodevelopmental functions, such as cell survival, maturation, and differentiation. Furthermore, it has been suggested that the smallest particles may enter endocrine glands and alter the endocrine system. Hypothyroxinemia in the first trimester, when neuronal migration begins, raises the risk of poor neuropsychological development in the fetus because it reduces the amount of maternal thyroxin (T4) available to the developing brain. It has been proposed that exposure to PM2.5 during pregnancy raises apoptotic protein levels at a critical juncture in brain development^[6]^.

Reactive oxygen species (ROS) damage cells via two mechanisms. Low-intensity ROS (superoxide, hydrogen peroxide) disrupt mitochondrial function, while high-intensity ROS (hydroxyl radicals, peroxynitrites) directly damage biomolecules. Excess ROS causes mitochondrial malfunction, which results in apoptosis or necrosis by depleting ATP or triggering proapoptotic proteins like Cytochrome C. Microglial activation and cytokine production speed up neurodegeneration, and elevated mitochondrial ROS (mtROS) also encourage neuroinflammation^[13]^.

Thermal stress is another critical mechanism. High temperatures can cause hyperthermia, leading to direct brain cell and tissues damage. Also, the inability of the human body to maintain homeostasis is influenced by thermal stress, which leads to a probable loss of neurotransmitter function and, thus, cognitive impairment^[14]^. Additionally, the stress of combating extreme weather changes can trigger or further deter mental health disorders such as post-traumatic stress disorder (PTSD), anxiety, and depression^[15]^.

Neurological health issues, furthermore, are tied to climate change-induced displacement and socio-economic disruptions. When sea levels rise, agricultural conditions change and extreme weather conditions are experienced, people can become displaced, thereby losing their social networks and becoming economically unstable and more stressed, some things that increase risk factors for mental health disorders^[16]^. The incessant stress connected with these disturbances may change how

### Current understanding of the relationship

Though the area is still in its early stages and needs more growth to reach greater depth, research on the effects of global warming on mental health is growing steadily. Therefore, although it has been established that several environmental factors underpinning climate change are responsible for various neurological diseases, we are yet to ascertain how. Research has increasingly highlighted the importance of the detrimental effects of air pollution and heat stress in adding to the burden of neurological disorders^[17]^.

Epidemiological research has found links between high pollution levels and cognitive decline, along with elevated risks for neurodevelopmental and neurodegenerative disease eases. For instance, in their study, Chen and Schwartz (2009) observed a lower cognitive function in older adults after having been exposed for a longer period to PM2.5 particles. In the same way, research by Heusinkveld *et al* (2016)^[18]^ found that air pollution might speed up neurodegenerative diseases through inflammatory routes.

Populations among susceptible individuals, such as the older section of the population and those with underlying health conditions, heat waves have noticeably increased hospital admissions related to neurological conditions.

Heat waves were linked to an increased likelihood of hospital admissions for mental disorders (F00-F79). In comparison to non-heat wave times, the relative risks for 1-day, 3-day, and 7-day heat waves were 1.04 (95% CI: 0.95–1.13), 1.15 (95% CI: 1.005–1.31), and 1.36 (95% CI: 1–1.90), respectively. Significantly higher admission rates were noted among men, the elderly, and the rural. According to subgroup analysis, admissions for mental retardation (F70–F79) and organic mental diseases (F00–F09) were more common during heat waves^[19]^.

The unavoidable consequences of the change of climate on mental health, such as anxiety, depression, or PTSD after extreme weather events or natural calamities, are widespread and well-known^[20]^.

Despite these developments, our knowledge needs more information. Over time, more studies must be undertaken on the impact of change of climate on neurological health to determine their causative agents. Furthermore, research should look into how we can combine various environmental stressors and find ways of mitigating or adapting to better protect brain health in an altered climatic environment.

## Emerging neurological challenges

### Increased incidence of heat-related illnesses and neurological conditions

**Figure 3.**
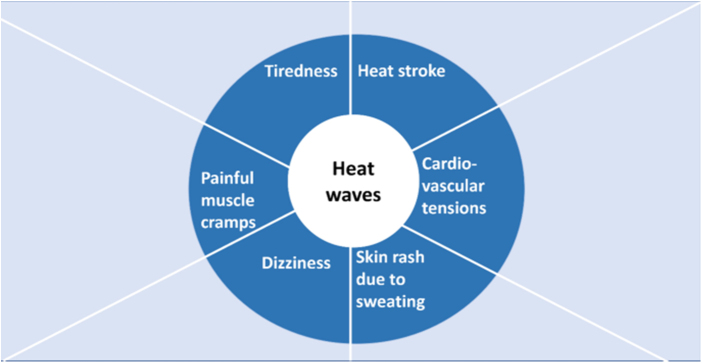
Effects of heat waves

Neurological health has a clear connection with the global rise in temperatures and the growing occurrence of intense heat waves because of climate changes. Heat-related illnesses, like heat exhaustion, are now common and are associated with damage right inside the mind. Heat stroke, in particular, can be a severe medical condition in which the body’s mechanisms for regulating temperature fail, and its core temperature rises excessively, leading to brain malfunction later. It may first seem like confusion or mild lightheadedness, but then it can worsen, causing seizures, coma, or even death due to heat stroke in the long run^[21]^ (Fig. 3).

Individuals with multiple sclerosis (MS) are exceptionally vulnerable to heat, which can worsen symptoms in a condition known as Uhthoff’s phenomenon. The condition involves temporary worsening neurological manifestations resulting from increased body temperature that disrupt nerve conduction. People with existing neurological diseases may have more severe problems when the weather is too hot^[16]^. Heat waves increase the probabilities of ischemic and hemorrhagic strokes by causing dehydration, alterations in blood viscosity, and vascular tension^[22]^.

Moreover, extreme heat can impact one’s cognitive functioning and state of mind. Some cognitive deficits have been observed, such as trouble understanding, memory loss, and weighing options. They may, in turn, affect output and general regard for life in totality^[23]^. Anxiety, depression, and worsening of schizophrenia, among other mental illnesses, are also related to sun heat; therefore, such conditions as these record a rise in hospital admissions during periods of intense heat^[24]^.

### Changes in disease patterns and distribution

Climate change is changing the patterns and distribution of several diseases with significant neurological effects. Diseases that are vector-borne, and are transmitted by mosquitoes and ticks, are finding their way into new regions because increased heat levels and altered rainfall patterns make them more comfortable for these vectors. Diseases such as Zika Virus, Lyme disease, and West Nile Virus, which can cause severe neurological complications, are increasing in incidence in areas that have never been affected before.

Infections like West Nile that mosquitoes can transmit can lead to conditions like encephalitis and meningitis, with long-term neurological sequelae such as cognitive dysfunction, muscle weakness, and movement disorders. As temperatures rise, the regional variety of the mosquito species that transmit the West Nile virus is expanding, leading to more frequent outbreaks in temperate regions^[25]^. Lyme disease which is caused by Borrelia burgdorferi, spreads by ticks. It causes multiple neurological issues, including signs of meningitis, cranial neuropathy, and radiculopathy^[26]^.

Like other mosquito-borne illnesses, Zika has caught the attention of the entire world because babies whose mothers have been infected with it develop microcephaly and other severe brain abnormalities that result from this infection. Consequently, altering the areas of Aedes mosquitoes leads to changes in the transmission patterns of the Zika virus, which may heighten the risk of it being introduced to new regions and lead to outbreaks^[27]^.

### Neurological health and extreme weather conditions

Climate change-induced increased and severe, frequent hurricanes, floods, and wildfires, such as extreme weather occurrences, significantly impact neurological health. Neurological consequences immediately after the fact constitute one way these disasters come about: toxins exposure or direct injury, while others are borne by psychological stress.

Hurricanes and high-water levels are potential causes of traumatic brain injuries (TBIs) when there is direct exposure to physical violence. Chaotic and dangerous situations bring about an elevated danger for one’s head: getting hurt by waste matter, falling, and also performing vehicle-related collisions. These injuries lastingly disorganize mind processes, remembrance, or feelings^[28]^. In addition, flooding may cause the contamination of water supplies with neurotoxic substances like heavy metals and pesticides that negatively affect the brain’s health^[29]^.

Environmental pollutants in drinking water including Acrylamide exposure, from contaminated water or plastered pipes can cause neurotoxic effects like tremors, neuropathy, sensory ataxia, hallucinations, and memory impairment. It also increases the risk of brain tumors. Certain pesticides (e.g., carbamates, organochlorines, organophosphates) cause irreversible brain damage and are linked to neurodegenerative diseases. Alumina, used in water treatment, elevates aluminum levels, which are associated with Alzheimer’s disease^[3]^.

Due to their exposure to smoke and particulate matter, wildfires present large risks to brain health. The inhalation of wildfire smoke is associated with higher levels of respiratory and cardiovascular hospitalization and neurological effects. Particulates in bushfire smoke can make neuroinflammatory disorders worse and promote dementia^[30]^.

Furthermore, the most important factor to consider is the psychological effect of weather changes on a person. Such things as trauma or stress can accompany these types of disasters and result in various mental conditions like PTSD, anxiety, or depression, among others. Additionally, prolonged psychological distress in survivors of such incidents can alter their brain activities and general mental well-being^[31]^. The disruption of social networks, loss of homes and livelihoods, and the ongoing stress of rebuilding can further compound these effects.

## Potential impacts on brain health

### Neuroinflammation and oxidative stress are fundamental processes

Neuroinflammation and oxidative stress are fundamental processes through which disruptions to climate can impact brain health^[32]^.

Oxidative stress (OS) is a condition resulting from the uncontrolled production of reactive oxygen species (ROS). Increased-ROS production shifts the redox balance of the cell towards the oxidative state, leading to cellular dysfunction and death. ROS can be produced as by-products due to the metabolism of environmental chemicals.

Neuroinflammation is the inflammatory response of the CNS cells (astrocytes and microglia) to stress, trauma, and disease. These factors cause the activation of microglia resulting in the discharge of several inflammatory and cytotoxic components^[33]^.

Consequently, it is essential to maintain body balance within the nervous system and defend it against any form of damage or disease. Nonetheless, chronic neuroinflammation can have adverse effects since it causes the development of different neurological disorders like multiple sclerosis (MS), Parkinson’s disease (PD), and Alzheimer’s disease (AD)^[32]^.

There are many ways through which neuroinflammation is worsened by climate change. One major cause is the rise in air pollution, which contains particulate matter (PM2.5), Nitrogen Oxide (NO), and ozone. The blood-brain barrier is permeable by these pollutants, which in turn trigger microglial cell activation, resulting in chronic inflammation^[11]^ – moreover, increased temperatures due to climate change directly cause inflammation in the brain. Pro-inflammatory cytokines and heat shock protein production can be increased by heat exposure, leading to neuron impairment and the wearing away of the brain^[34]^.

Neuroinflammation is closely linked to oxidative stress, a condition where an imbalance of reactive oxygen species (ROS) damages cells. ROS can harm lipids, proteins, and DNA, leading to cellular dysfunction and, ultimately, neuronal death. Oxidative stress can increase in the brain due to exposure to polluted air and heat. There are particulate matters that, when inhaled, induce oxidative stress and inflammation that eventually lead to neurodegeneration^[11]^. Again, it may not be known that these ways of destabilizing cellular homeostasis include exposure to extreme temperatures, thus increasing the production of ROS and causing oxidative damage^[35]^.

### Neurological consequences of air pollution and exposure to toxins

One significant neurological consequence of pervasive environmental health hazards is contamination of air. Observation shows that contact with nitrogen dioxide (NO2), PM2.5, and sulfur dioxide (SO2) for a long deterioration period brings about deterioration of cognitive abilities and subsequent risk of contracting Alzheimer’s disease, among other types of dementia^[3]^. Air pollution affects the brain in two main ways: direct and indirect pathways. Pollutants inhaled could get into the blood vessels and then find their way to the brain, which may lead to inflammation as well as oxidative reactions. Meanwhile, the body may release inflammatory mediators because of pollution-induced systemic inflammation, thereby affecting blood-brain barrier integrity and leading to neuronal loss and cognitive decline^[11]^. Other environmental toxins such as heavy metals (like lead, mercury, and arsenic) and pesticides (particularly organophosphates) could also have severe implications for human health in terms of neurology, including cognitive impairment, developmental problems in young children, and increased chances of getting neurodegenerative diseases^[36]^.

### Implications for cognitive function and mental health

Change of climate and its related factors profoundly impact cognitive function and mental health. These cognitive functions may be negatively affected due to exposure to air pollution or heat stress, among others. For instance, children living in polluted areas have reduced neural development and lower cognitive performance^[37]^. Altering similarly, heat effects can make blood flow within the brain, thereby leading to dehydration risks, which reduce people’s ability to think clearly or make good choices^[23]^. Climate change significantly affects people’s mental health. For example, depression, PTSD, and anxiety may arise among individuals due to severe weather events like hurricanes as well as other calamities such as floods and fires^[31]^.

## Vulnerable populations

### Children and developmental vulnerabilities

Children’s developing bodies and brains are highly susceptible to climate-related neurological disturbances. Cognitive and neural impairments can be permanent if children are exposed to environmental toxicants during crucial stages of growth. For instance, reduced IQ, attention deficits, and an enhanced likelihood of Autism Spectrum Disorders (ASDs) have all been linked to prenatal and early exposure to pollution of air^[37]^. Pollutants such as lead, mercury, and particulate matter pollute and disrupt neurodevelopmental processes in the developing brain, where they can cause lifelong impairments^[36]^.

High temperatures can also be hazardous to kids due to their underdeveloped thermoregulatory systems; hence, they are more vulnerable to heat stroke or dehydration. The situation could even escalate to conditions like fever and loss of senses that cause severe problems in the brain. Additionally, extreme weather events increase children’s risk of developing anxiety, depression, and behavioral disorders^[38]^.

### Elderly and age-related neurological risks

**Figure 4.**
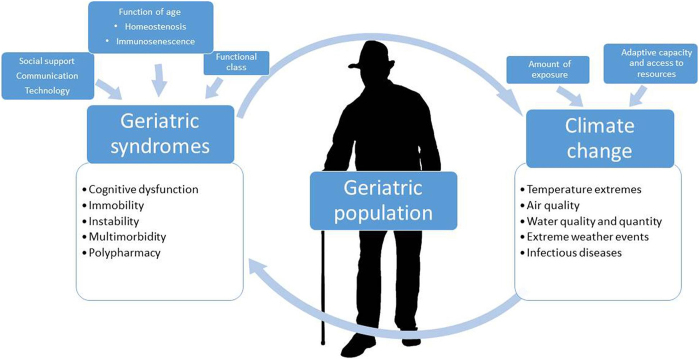
Effects of climate changes on geriatric population.

Elders represent one more group particularly prone to the neurological effects of the change of climate, as they have experienced other types of injuries before. For instance, due to age-related reductions in physiological resilience and learning capacity, older adults are more vulnerable to being affected by environmental factors. For example, the elderly can be especially susceptible to heat waves since they usually cannot control their bodies’ temperatures and are more prone to heat-triggered health issues such as sunstroke and lack of water. Such disorders exacerbate pre-existing neurological disorders and result in acute cognitive deficits^[39]^ (Fig. 4).

Additionally, there is a correlation between air pollution and hastened cognitive decay, as well as a higher probability of older people contracting Alzheimer’s disease and Parkinson’s disease, which are degenerative diseases of the brain. Referring to pollutants over time thus results in chronic inflammation of nerves and strain emanating from free radicals, culminating in damage to cells, hence worsening the situation in those with such disorders^[3]^. Older people are expected to experience more mental health issues related to climate change, most commonly anxiety, depression, or developing posttraumatic stress disorders, especially after facing severe climatic conditions^[20]^.

### Socioeconomic factors influencing vulnerability

Socioeconomic factors heavily influence the neurological effects of climate change. Access to health care is often limited for people from poorer backgrounds, who reside in areas with high pollution levels and may need more resources to mitigate extremely harsh weather conditions. These may make the neurological dangers connected to climatic conditions even worse.

For instance, regions with people who do not have much money tend to live next to industries that release many gases into the air, thus attracting more dangerous nerves and toxins. Moreover, the lack of cooling centers or air conditioning in low-income societies during high temperatures may result in higher chances for heat-caused diseases with neurological outcomes^[40]^. Low socioeconomic status reacting to restricted accessibility to resources may also trigger mental health issues such as more cases of anxiety, depression, and other mental disorders^[41]^.

Socioeconomic disparities also influence access to education and healthcare, thus affecting mitigation capability and adaptability to the neurological effects of climate change. Such people will likely need more access to relevant information concerning risks and protective measures related to climate change and, hence, become more exposed to risks^[36]^.

## Vector-borne diseases and neurological health

### Impact of changing disease vectors on neurological health

Climate change significantly impacts the distribution, seasonality, and abundance of disease vectors like mosquitoes and ticks, which heavily affect neurological public health. With increases in temperatures and alterations in rainfall, mosquito populations and other vectors initially confined within some regions have been moving outwards, thereby heightening their transmission risks from one population group to another that had hitherto experienced none.

Rising temperatures are expanding the habitats of Aedes mosquitoes (Chikungunya), which transmit diseases such as Dengue fever, Zika virus, and Chikungunya. Consequently, we have seen these diseases become more widespread than before, particularly in North America and Europe, where they were less familiar or even unknown before^[42]^. Similarly, Ixodes ticks, which carry Lyme disease, are moving to higher latitudes and elevations due to warmer winters and, thus, extended summers, leading to increased Lyme disease risk at novel sites^[43]^.

This shift of vector spread calls for significant public health challenges, such as the need to incorporate new territories within the surveillance and control regimes. An increase in exposure to VBDs may mean more neurological complications associated with them, which can touch a broader section of your community as a whole^[44]^.

### Neurological consequences of diseases that are vector-borne

Vector-borne diseases often lead to severe neurological disorders, which range from simple cognitive deficits to fatal conditions. Zika Virus, Lyme disease, and West Nile Fever are some of the most notable vector-borne diseases with neurological impacts.

#### West nile virus

Fever, fever. Mosquitoes of Culex largely transmit West Nile virus (WNV), which can give way to West Nile fever, sometimes progressing to neuroinvasive diseases in severe instances. Consequently, there could be encephalitis, meningitis, or acute flaccid paralysis cases, among others. Instead, survivors may suffer chronic cognitive malfunction, muscle weakness, or even motor deficits as neurological sequelae^[45]^.

#### Lyme disease

The causative agent of Lyme disease is a bacterium that is transmitted through the Ixodes tick called Borrelia burgdorferi, which can lead to Lyme neuroborreliosis. The condition is evidenced through lymphocytic meningitis, cranial neuropathy, and radiculo-neuropathy. Chronic neurological syndromes that endure beyond antibiotic regime include memory problems, fatigue, and pain^[46]^.

#### Zika virus

Aedes mosquitoes that transmit Zika have sparked global concern because they are linked with a congenital disability called ‘Zika virus disease. ‘If an infected mother, then carries a child, he/she may end up having some neurological disorders like brain reduction. At the same time, there might be Guillain-Barré Syndrome (GBS) in adults due to this same sickness, such as the immune system attacking nerves and producing paralysis and muscular weakness^[47]^.

The neurological impacts of these, as well as various other vector-borne diseases, emphasize the necessity of fully bundled tactics aimed at reducing the spread of these parasites or pathogens while controlling health states among those who have been affected.

### Strategies for prevention and control

Things like preventing and controlling infections like malaria or yellow fever have required us to combine several things, like vector control and individual protection methods, with public health interventions.

#### Vector control

Genetics: Reducing insect populations is important to controlling diseases. This would involve eliminating their breeding grounds, using chemicals, or turning to organisms that feed on them^[48]^. In the same vein, modifications have been made using genetics, as indicated by some kind of mosquito, with genes that prevent it from reproducing or causing any pathogens transmission^[49]^.

#### Public health interventions

Public health is key in monitoring and controlling VBDs; that is, it intervenes early enough through surveillance programs, which trace vectors’ populations and disease incidence, for appropriate response and containment of the outbreak. Public health is also critical in publicizing preventive measures among community members, such as applying insect repellent or wearing protective gear^[50]^.

#### Individual protective measures

There are several ways individuals can use to protect themselves from vector-borne diseases. This may involve insect repellent, fitting window and door screens, and using long-sleeved clothes to reduce the skin area exposed to vectors. Available vaccination is also an important preventive measure. For example, vaccines against Japanese encephalitis or yellow fever infections help lower the probability of contracting these infections by vectors.

## Mental health implications

### Psychological impacts of climate change on neurological health

Climate change alters mental health significantly, thus influencing neural health indirectly through either immediate or long-term environmental stress factors. An example of an immediate stressor includes hurricanes, among others, since they lead to instant trauma – time and atmosphere. However, when we talk about sustained stressors that are usually extended over time, such as increasing temperatures in the atmosphere, they cause people to be anxious, always leading to depression. *Climate change* is a constant problem that continuously causes persistent stress and psychological burden^[20]^. As such, neural impairments are evident when one is highly stressed from one point to another due to the release of cortisol in life-threatening situations, which destroys brain structures such as the hippocampus and amygdala, resulting in cognitive damage that will last a long time, thus making people vulnerable to mental health illnesses^[51]^. In addition, chronic environmental stress promotes neuroinflammation and oxidative stress, which in turn can contribute to neurodegenerative conditions such as Alzheimer’s disease and other forms of dementia^[52]^.

### Risk of depression, anxiety, and stress-related disorders

The increased risk of anxiety, depression, and stress-related disorders due to climate change is well-documented. Many individuals experience ongoing anxiety about the future, often referred to as “eco-anxiety,” characterized by chronic worry and helplessness regarding the environment and the impacts of the change of climate^[53]^. Extreme weather events worsen climate change, causing displacement, loss, and increased depression. Also, living in a continually changing and hazardous environment can create extensive pressure that causes depression^[20]^. Survivors of climate-related disasters face a high prevalence of stress-related disorders. This is due to distress arising from the loss of family members, property, and houses, leading to intense psychological tension and permanent brain damage that constrains cognitive functioning and the management of emotions^[31]^.

### Importance of mental health support in climate change adaptation

As climate change has some severe implications for mental health, it is significant to adjust mental health assistance in treatment approaches. Recovery process: To minimize emotional abnormalities, support the recovery process, and build resilience, the healthcare provider should ensure mental health services to the affected individuals. These programs can significantly aid those who desire to overcome trauma and manage stress through counseling sessions and educational materials^[54]^. Hence, Preparation for a public health disaster and a response strategy should integrate mental well-being protocols such as the prompt responsiveness of mental health advisory services amidst unavoidable weather conditions and amendments in policies to minimize stressors of the environment^[53]^.

## Global health policy and climate change

### Role of international organizations in addressing neurological consequences

The **World Health Organization (WHO)** exercises its authority in highlighting the impact of climate shift, linked to mental health and neurological problems. It finally makes strategies and assists different countries to form and regulate mitigation precautions addressing healthcare. Additionally, their objective is to minimize health problems related to the change of climate, consequently promoting and developing advanced health systems capable of managing illnesses caused by extreme weather conditions^[55]^. Furthermore, suggests that mental health considerations and mental health and psychosocial support (MHPSS) approaches should be integrated into health systems to enhance resilience. Incorporating MHPSS into climate change policies like Health National Adaptation Plans (HNAPs), Long-Term Low-Emission Development Strategies (LT-LEDS), and Nationally Determined Contributions (NDCs).Establishing cross-sectoral MHPSS coordination mechanisms. Promoting communication between MHPSS services and climate action sectors. Encouraging community involvement in climate adaptation and mitigation^[56]^. However, a significant policy gap lies in the *implementation* of these recommendations at the national level. Many countries, particularly low- and middle-income nations, lack the resources and infrastructure to effectively integrate MHPSS into their health systems and climate change adaptation strategies. Feasibility hinges on increased financial and technical assistance from international bodies and developed nations^[55]^.

#### Intergovernmental panel on climate change (IPCC)

The main object of the IPCC is to formulate a charter that suggests a thorough understanding of the subject of climatic change, and its futuristic challenges. It also considers the health effects of climate change, such as neurological health, and advises policymakers worldwide. In shaping global warming laws, intergovernmental adaptation programs on health depend on the findings from the reports submitted by the IPCC (Intergovernmental Panel on Climate Change, 2014). Their reports inform policymakers worldwide, shaping the scientific basis for adaptation and mitigation efforts. While the IPCC meticulously documents the evidence, a challenge remains in translating these scientific findings into concrete and actionable policy measures, specifically concerning neurological health. Future IPCC reports could benefit from more focused analyses and recommendations regarding the neurological consequences of specific climate change scenarios, thereby providing more targeted guidance for policymakers^[57]^.

#### United Nations framework convention on climate change (UNFCCC)

The UNFCCC is supporting international collaboration to address climate change and its various impacts. It advocates for policies that tackle the health consequences of climate change, focusing on neurological ones. It also assists countries with finance and technology transfer to address the impact of climate actions on health. While the UNFCCC advocates for policies that tackle the health impacts, including neurological ones, and supports developing countries through finance and technology transfer, the specific allocation of resources and the prioritization of neurological health within broader climate change agendas often remain unclear. Greater emphasis within UNFCCC frameworks on the neurological burden of climate change and dedicated funding mechanisms could significantly enhance global action in this area^[57]^.

### Policies for climate change adaptation and mitigation

#### Health adaptation plans

Countries in various stages of development have begun developing health plans to combat the effects of change of climate on human health, including neurological health. These could incorporate the National Heatwave Plan of France and the Climate and Health Program of the United States to protect public health from threats posed by the change of climate. It includes performing vulnerability assessments, implementing early warning systems, and establishing robust healthcare systems. However, many existing plans lack specific attention to neurological vulnerabilities, such as increased stroke risk during heatwaves or the mental health impacts of extreme weather events. Future iterations of these plans need to explicitly incorporate strategies for neurological preparedness and response, including specialized training for healthcare professionals and accessible support services for affected populations^[58]^.

Increase knowledge of and response to the effects of climate change on brain health utilizing innovative research, interdisciplinary cooperation, and calculated advocacy. Combining knowledge from environmental science and neuroscience to look into how climate-related issues impact mental health and neurological function. Enhance global public health outcomes by offering well-informed, empirically supported solutions to these new issues. Multidisciplinary cooperation between neurologists, climate scientists, and legislators to guarantee that healthcare systems are prepared to handle these new issues^[59]^. Reducing dangers by employing better infrastructure, and public health initiatives. To lessen the neurological effects of climate change, a proactive strategy that emphasizes education, early interventions, and community-based resilience techniques is essential^[12]^. Conduction of conferences like the “Hot Brain Conference on Climate Change and Neuroscience” represents initiatives to deal with these intricate relationships^[60]^. However, sustained funding for such interdisciplinary research and the translation of findings into public health interventions remain critical challenges. Policy supports for collaborative research networks and dedicated funding streams for climate change and neuroscience research are essential to bridge this gap. Furthermore, integrating neurological expertise into climate change policymaking processes is crucial to ensure that adaptation and mitigation strategies adequately consider brain health^[59,60]^.

#### Mitigation policies

Hence, policies that minimize the emission of greenhouse gases and reduce the threat of change of climate and its health effects are vitally important. Resultant public health problems from global warming can be alleviated by shifting from nonrenewable sources of power; another way is to increase energy efficiency and lower production from the transport and agricultural sectors. Policy coherence across energy, transportation, agriculture, and health sectors is crucial to maximize these co-benefits^[61]^.

### Collaborative efforts to protect neurological health on a global scale

#### International research collaboration

Collaborating on an international level is needed among researchers if they are to comprehend the complex relationships linking climate shifts to neurological problems. This can be achieved by conducting joint studies on large populations, sharing ideas, and adopting similar research procedures. Global Consortium on Climate and Health Research is devoted to fostering international cooperation for investigation and creating worldwide capacity for solving climate-driven health challenges. However, ensuring equitable participation and data sharing across countries, particularly between high- and low-income nations, remains a challenge. Policies that promote equitable research partnerships and facilitate the dissemination of findings are crucial^[62]^.

#### Global health initiatives

Diseases Global health programs like the Global Health Security Agenda aim to boost the global health systems to mitigate, identify, and stop threats from various diseases, including climate change-related ones. With these programs, other countries are advised to come together and share their resources to boost health security in the entire world and be able to shield their neurological health from the harmful effects that are brought about by these climate changes ^[58]^.

#### Policy integration

Integrating climate change considerations into health policies is critical to protecting people’s brain health. Government, international agencies, and civil society must lead by considering mitigation and adaptation aspects. To safeguard the human population from these disasters, preparedness plans must also exist within existing healthcare systems. This includes developing preparedness plans within healthcare systems to address the neurological consequences of climate-related events and ensuring access to mental health support for affected populations. Strengthening cross-sectoral collaboration between health, environment, energy, and social welfare ministries is crucial for a holistic and effective response^[63]^.

## Research gaps and recommendations

### Areas needing further research and exploration

1. **Longitudinal Studies on Chronic Neurological Impacts**: While cross-sectional studies and ecological analyses have established associations, there is a critical need for longitudinal studies with extended follow-up periods to elucidate the chronic neurological consequences of prolonged exposure to climate change-related factors. This includes investigating the cumulative effects of chronic low-level exposure to air pollutants and the long-term impact of recurrent heat waves on cognitive function and the risk of neurodegenerative diseases like Alzheimer’s and Parkinson’s^[3]^. Such studies should employ robust exposure assessments and repeated neurological evaluations to establish temporal relationships and dose-response curves.
2. **Vulnerability of Specific Populations**: Further in-depth research is required to fully characterize the heightened vulnerability of specific populations. Studies focusing on the neurological development of children exposed to climate-related stressors *in utero* and during early childhood are essential to understand potential lifelong impacts on cognitive function and neurobehavioral disorders^[36]^. Similarly, prospective studies on older adults are needed to disentangle the interaction between aging, pre-existing conditions, and climate-related exposures in the development and progression of neurodegenerative diseases and cerebrovascular events. These studies should consider genetic predispositions and other individual susceptibility factors.
3. **Mechanistic Investigations**: A deeper understanding of the underlying biological mechanisms linking climate change to neurological disorders is crucial for developing targeted interventions. Future research should prioritize investigating the roles of neuroinflammation, oxidative stress, and disruptions in the neurovascular unit in mediating the effects of air pollution, heat stress, and vector-borne diseases on the brain^[37]^. Utilizing *in vivo* and *in vitro* models, coupled with human biomarker studies, can provide insights into the molecular pathways involved. Furthermore, research exploring the interplay between environmental exposures and genetic susceptibility in the development of neurological diseases is warranted.
4. **Mental Health Consequences of Climate Change**: While the immediate psychological impacts of extreme weather events are increasingly recognized, more research is needed on the long-term mental health consequences of chronic climate stress, including climate anxiety, eco-grief, and the exacerbation of pre-existing mental health conditions^[53]^. Studies should explore the neurobiological underpinnings of these conditions and evaluate the effectiveness of different psychosocial interventions in building resilience.
5. **Impact of Climate Change on Vector-Borne Neurological Diseases**: Research should focus on predictive modeling of the changing geographic distribution and seasonality of vector-borne diseases with neurological sequelae, considering various climate change scenarios^[44]^. Furthermore, studies are needed to understand the long-term neurological outcomes in survivors of these infections and to develop effective strategies for prevention, early diagnosis, and management.

### Methodological challenges in studying neurological consequences

1. **Standardization of Data Collection and Exposure Assessment**: A significant methodological challenge lies in obtaining high-quality, comparable data across diverse geographical regions. Establishing standardized protocols for data collection, including uniform definitions of neurological outcomes and consistent methods for assessing exposure to climate-related factors (e.g., air pollution monitoring, heatwave indices, vector surveillance), is essential for improving the rigor and comparability of research findings^[64]^. Utilizing remote sensing technologies and geographic information systems (GIS) can enhance the spatial and temporal resolution of exposure data.
2. **Multidisciplinary and Integrated Approaches**: Understanding the complex interplay between climate change and neurological health necessitates multidisciplinary collaborations involving neurologists, epidemiologists, environmental scientists, climatologists, toxicologists, and social scientists^[65]^. Future research should adopt integrated study designs that simultaneously consider multiple environmental stressors and their interactions, as well as individual and societal factors. This includes incorporating ecological momentary assessment (EMA) techniques to capture real-time exposure and symptom data.
3. **Addressing Confounding Factors**: Disentangling the specific effects of climate change-related factors on neurological health from other potential confounders, such as socioeconomic status, access to healthcare, lifestyle factors, and pre-existing conditions, poses a significant challenge^[54]^. Future studies should employ advanced statistical methods, including multivariate regression models, mediation analysis, and causal inference techniques, to carefully control for these confounding variables and establish more robust causal relationships.
4. **Leveraging Big Data and Artificial Intelligence**: The increasing availability of large-scale health and environmental datasets presents opportunities for utilizing big data analytics and artificial intelligence (AI) to identify patterns, predict risks, and personalize interventions related to climate change and neurological health. Machine learning algorithms can be employed to analyze complex interactions between environmental exposures, genetic factors, and neurological outcomes^[66]^.
5. **Development of Early Detection and Biomarkers**: Research should focus on identifying early biomarkers of climate-related neurological damage. This could involve investigating changes in neuroimaging, cerebrospinal fluid, blood-based biomarkers, or electrophysiological measures in individuals exposed to climate stressors. The development of sensitive and specific biomarkers would facilitate early detection, risk stratification, and the evaluation of preventive interventions^[55]^.
6. **Intervention and Adaptation Research**: Beyond understanding the problem, future research must focus on developing and evaluating effective interventions and adaptation strategies to mitigate the neurological impacts of climate change. This includes evaluating the effectiveness of heatwave early warning systems, air pollution reduction strategies, vector control programs, and mental health support services in protecting brain health. Community-based participatory research approaches can ensure the relevance and feasibility of interventions in diverse settings^[54]^.

## Discussion

This review synthesizes the growing body of literature examining the intricate relationship between climate change and neurological health. Our analysis underscores the multifaceted ways in which a changing climate, characterized by rising temperatures, altered weather patterns, and increased environmental pollution, poses significant threats to brain function and increases the risk of various neurological disorders.

The evidence presented highlights that environmental factors exacerbated by climate change, such as extreme temperatures and air pollution, directly contribute to neurological morbidity. Exposure to air pollutants like PM2.5, NO_2_, and SO_2_ is consistently linked to cognitive decline and an increased risk of neurodevelopmental and neurodegenerative diseases, aligning with findings from studies by Chen and Schwartz (2009) and Heusinkveld *et al* (2016)^[11,18]^. The proposed mechanisms involve the ability of these pollutants to cross the blood-brain barrier, inducing neuroinflammation and oxidative stress, which are central to the pathogenesis of many neurological conditions^[11]^.

Furthermore, our review emphasizes the direct impact of rising temperatures and heat waves on neurological health. Heat-related illnesses, ranging from heat exhaustion to severe heat stroke, can lead to direct brain damage and exacerbate pre-existing neurological conditions, as seen in the vulnerability of individuals with multiple sclerosis^[16,21]^. The epidemiological data, such as the study by Louis *et al*^[9]^. in China and South Korea, which linked temperature variability and heat waves to increased stroke incidence and cerebrovascular disease burden, underscores the significant neurological consequences of extreme heat. The observed increase in hospital admissions for mental disorders during heat waves^[19]^ further highlights the broad impact of thermal stress on brain health.

Beyond direct environmental stressors, climate change also indirectly affects neurological health through alterations in disease patterns and the increased incidence of vector-borne diseases. The expansion of the geographical range of disease vectors like mosquitoes and ticks, driven by changing temperatures and rainfall patterns, is leading to the emergence of diseases such as Zika virus, West Nile fever, and Lyme disease in previously unaffected regions^[42,43]^. The neurological sequelae of these infections, including meningitis, encephalitis, cognitive dysfunction, and developmental abnormalities, present a growing public health challenge^[45]^,^[46,47]^.

Our review also identifies neuroinflammation and oxidative stress as fundamental pathways through which climate-related disruptions impact brain health^[32]^. Increased exposures to air pollutants and extreme temperatures can trigger and exacerbate these processes, leading to neuronal damage and contributing to the development and progression of neurodegenerative diseases^[11]^,^[34,35]^. The intricate interplay between environmental stressors, neuroinflammation, and oxidative stress underscores the complexity of the relationship between climate change and neurological health.

Vulnerable populations, including children, the elderly, and those from lower socioeconomic backgrounds, are disproportionately affected by the neurological consequences of climate change. Children’s developing nervous systems are particularly susceptible to the harmful effects of environmental toxins and extreme temperatures, potentially leading to long-term cognitive and neurodevelopmental impairments^[37,38]^. The elderly, with their reduced physiological reserves and increased prevalence of pre-existing conditions, face a higher risk of heat-related illnesses and accelerated cognitive decline due to air pollution^[39]^. Socioeconomic disparities further exacerbate these vulnerabilities by limiting access to resources, healthcare, and protective measures^[40,41]^.

While the current understanding of the neurological impacts of climate change is evolving, significant gaps remain. Further research is needed to elucidate the precise mechanisms by which various environmental stressors interact to affect brain health, identify specific biomarkers for early detection of climate-related neurological damage, and develop effective mitigation and adaptation strategies. Longitudinal studies examining the long-term neurological consequences of chronic exposure to climate-related stressors are crucial. Moreover, interdisciplinary collaborations involving neurologists, environmental scientists, public health experts, and policymakers are essential to address this complex and growing public health challenge.

## Conclusion

In conclusion, this review provides a comprehensive overview of the current knowledge regarding the neurological impacts of climate change. The evidence strongly suggests that a changing climate poses a significant and growing threat to brain health across various populations. Addressing this challenge requires a concerted effort to mitigate climate change, develop targeted interventions for vulnerable groups, and promote further research to enhance our understanding of the intricate links between our environment and our neurological well-being. Climate change poses a serious risk to neurological health, constraining urgent policy intervention. This review highlights complex interplay between environmental factors and brain health can be by stressing the urgent need for targeted research on neuroinflammation, oxidative stress, and the lifelong consequences of air pollution and extreme weather events. Working together to enhance data sharing is a start to dealing with these issues. Begin with developing targeted interventions for at-risk groups. Furthermore, you can provide mental health support in climate change adaptation strategies. Mitigating climate-driven neurological diseases like Alzheimer’s and stroke demands immediate global policy action, enhanced public health strategies, and strengthened interdisciplinary collaboration.

## Data Availability

The data sets for this study can be availed by the reader through the corresponding author.
